# Evaluation of a Novel Handoff Communication Strategy for Patients Admitted from the Emergency Department

**DOI:** 10.5811/westjem.2017.9.35121

**Published:** 2018-02-08

**Authors:** Christopher J. Smith, Russell J. Buzalko, Nathan Anderson, Joel Michalski, Jordan Warchol, Stephen Ducey, Chad E. Branecki

**Affiliations:** * University of Nebraska Medical Center, Department of Internal Medicine, Omaha, Nebraska; † Children’s Hospital & Medical Center, Omaha, Nebraska; ‡ George Washington School of Medicine & Health Sciences, Department of Emergency Medicine, Washington, District of Columbia; § Salt Lake Regional Medical Center, Department of Emergency Medicine, Salt Lake, Utah; ¶ University of Nebraska Medical Center, Department of Emergency Medicine, Omaha, Nebraska

## Abstract

**Introduction:**

Miscommunication during inter-unit handoffs between emergency and internal medicine physicians may jeopardize patient safety. Our goal was to evaluate the impact of a structured communication strategy on the quality of admission handoffs.

**Methods:**

We conducted a mixed-methods, pre-test/post-test study at a 560-bed academic health center with 60,000 emergency department (ED) patient visits per year. Admission-handoff best practices were integrated into a modified SBAR format, resulting in the Situation, Background, Assessment, Responsibilities & Risk, Discussion & Disposition, Read-back & Record (SBAR-DR) model. Physician handoff conversations were recorded and transcribed for the 60 days before (n=110) and 60 days after (n=110) introduction of the SBAR-DR strategy. Transcriptions were scored by two blinded physicians using a 16-item scoring instrument. The primary outcome was the composite handoff quality score. We assessed physician perceptions via a post-intervention survey.

**Results:**

The composite quality score improved in the post-intervention phase (7.57 + 2.42 vs. 8.45 + 2.51, p=.0085). Three of the 16 individual scoring elements also improved, including time for questions (70.6% vs. 82.7%, p=.0344) and confirmation of disposition plan (41.8% vs. 62.7%, p=.0019). The majority of emergency and internal medicine physicians felt that the SBAR-DR model had a positive impact on patient safety and handoff efficiency.

**Conclusion:**

Implementation of the SBAR-DR strategy resulted in improved verbal handoff quality. Agreement upon a clear disposition plan was the most improved element, which is of great importance in delineating responsibility of care and streamlining ED throughput. Future efforts should focus on nurturing broader physician buy-in to facilitate institution-wide implementation.

## INTRODUCTION

Patient care handoffs are a potentially vulnerable time for patient safety.^1^–^5^ Sub-optimal handoff communication is a common cause of medical errors^6^ and malpractice claims.^7^, ^8^ Handoff research has primarily focused on communication within a specialty or unit, such as those occurring at shift change. Recently, there has been increased focus on inter-unit handoffs, which occur when patients are transitioned between services, departments, or institutions.^9^ The inter-unit admission handoff between emergency physicians (EP) and inpatient providers is a particularly important example. The emergency department (ED) admission process involves changes in the healthcare team and physical location of the patient.^10^ Unstructured communication, inter-disciplinary conflict, patient throughput pressures, and uncertain assignment of responsibilities may further impede safe care transitioning from the ED to inpatient setting.^9^–^13^ Survey studies have found that one-third of physicians know of adverse patient events related to the admission handoff process.^10^, ^13^

Standardized handoff communication tools have been shown to improve outcomes for inter-unit handoffs,^6^ but they have not been widely implemented for admission handoffs. A survey of 750 physicians at 10 sites found that only 18% of EPs and internal medicine (IM) physicians used a standardized admission-handoff tool and only one-third of residents received handoff training.^14^ Although many handoff mnemonics exist in the literature,^15^–^17^ Situation, Background, Assessment, and Recommendation (SBAR) is the most commonly used,^18^ and is promoted by regulatory and professional organizations, including the Agency for Healthcare Research and Quality^19^ and Institute for Healthcare Improvement.^20^

In 2012 Beach et. al. published best practice recommendations for ED-to-inpatient handoff communication, including style, form and content. They suggested synchronous, two-way, closed-loop communication, with the goal of constructing a shared mental model of patient care between EPs and IM providers. Rather than rote recitation of data, it was suggested the content of handoff communication should focus on clinical judgment, diagnostic uncertainty, the patient’s clinical trajectory, pending tasks, and any patient- or system-level considerations that may impact care.^21^

A study in 2016 found that a standardized method of handoff for patients admitted to a geographically isolated, 35-bed community hospital from the ED resulted in fewer physician-reported “defective” handoffs;^22^ however, it is unknown if these findings are applicable to larger academic health centers that have unique complexities, including multiple admitting services with variable processes and trainees at various levels of training.^13^ The goal of this study was to pilot test a standardized process to improve admission handoff communication between EPs and IM physicians by integrating best-practice recommendations with a modified SBAR format, resulting in the Situation, Background, Assessment, Responsibilities & Risks, Discussion & Disposition, Read-back & Record (SBAR-DR) model. We hypothesized use of SBAR-DR would improve the quality of inter-unit handoff communication for patients admitted from the ED.

## METHODS

### Design and Setting

We conducted a mixed-methods, pre/post-study of admission handoff quality in conjunction with the implementation of the SBAR-DR strategy for admission handoff communication between EPs and IM physicians. The intervention took place at a 560-bed, Midwestern academic health center. The ED is a certified Level I trauma center with 60,000 patient visits per year, 15,000 inpatient admissions per year, and an emergency medicine (EM) residency program with 27 trainees. The IM service includes teaching and non-teaching teams, which admit approximately 6,000 patients per year, 60% of which are through the ED. The IM residency program has approximately 80 house officers. Prior to the intervention, there was no institutional standardized verbal or written handoff strategy. The project team consisted of resident and faculty physicians from EM and IM, as well as an educational expert specializing in training and performance improvement.

**Population Health Research Capsule**

What do we already know about this issue?

Inter-unit handoff from the ED to the inpatient setting is a vulnerable time for patient safety, but little research has investigated strategies to improve this process.

What was the research question?

How would a structured communication strategy impact the quality of handoffs between emergency physicians and internal medicine physicians?

What was the major finding of the study?

The admission handoff quality score improved following the intervention (7.6 vs. 8.5, p=.0085).

How does this improve population health?

Improving handoff practices has the potential to improve the care of the 12 million people/year admitted to the hospital from the ED.

### Verbal Handoff

**Figure 1:**
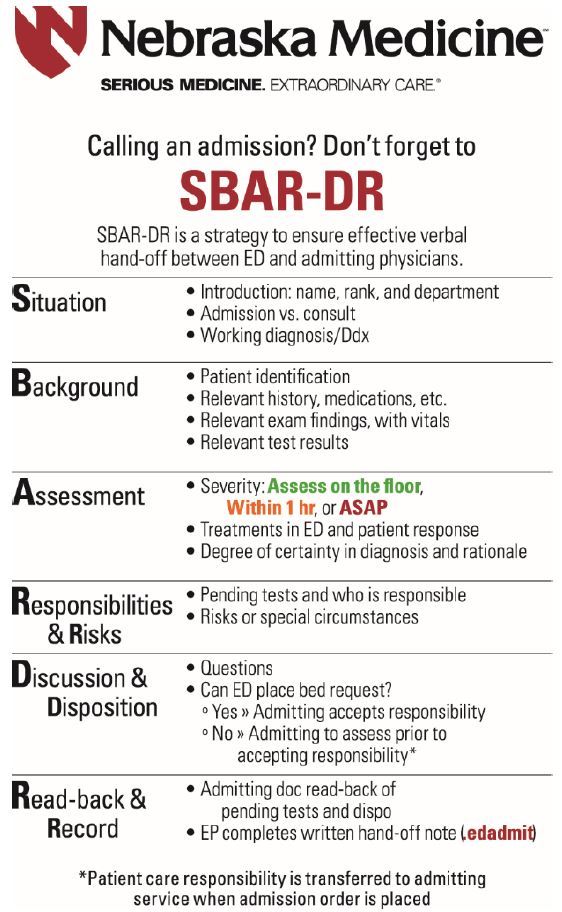
Situation, Background, Assessment, Responsibilities & Risks, Discussion and Disposition, Read-back & Record (SBAR-DR) format for admission handoffs.

The research team conducted iterative rounds of analysis to integrate admission handoff best practices^21^ into a modified SBAR format,^23^ resulting in the SBAR-DR model. Within each section there was clearly defined clinical information and communication guidance (Figure 1). For example, physicians were instructed to discuss severity of illness based on a three-tier system,^24^ ask clarifying questions, come to explicit agreement on the disposition plan, and use closed-loop communication. The transfer of patient care responsibility from EP to IM physician was linked to placement of an admission order, so as to remove ambiguity. Finally, EPs were asked to create a written handoff with the electronic health record (EHR) using an admission handoff template, further described below.

### Written Handoff Template

An admission handoff template was created within the EHR to supplement verbal handoff. The template was designed as an editable macro that could quickly be imported into the EP note. The template included headings for working diagnosis, description of the ED course, and a drop-down menu to list potential risks to patient care (e.g., prolonged boarding times). Data on pending tests and medications administered were automatically imported into the template. A definitive assignment of care responsibilities was documented at the end of the note, along with pager numbers for the appropriate admitting service to facilitate communication with ED nursing and ancillary staff. The EP completed the handoff note immediately after completion of the verbal admission handoff.

### Education

The research team developed an educational session that included a discussion of admission handoff best practices, a review of internal handoff data, and introduction to the verbal and written elements of the SBAR-DR model. The training included review and group discussion of two videos, one demonstrating poor handoff communication and one demonstrating high-quality communication using SBAR-DR.^25^ Resident and faculty physicians in EM and IM underwent training at required meetings in the two weeks prior to the introduction of the SBAR-DR process. Each session took approximately 30 minutes to complete. To reinforce the training, badge and pocket cards illustrating the SBAR-DR format were given to all participants. SBAR-DR posters were placed in the ED near recorded phone lines and in IM physician work rooms where they typically received admission handoff calls.

### Data Collection

#### Handoff recordings

Admission handoff conversations were recorded from two labeled ED telephone lines using a HIPAA-compliant online recording program for 60 days prior to (January 21 – March 21, 2015) and 60 days following (April 9 – June 7, 2015) the implementation of the SBAR-DR process. Participants were emailed consent cover letters prior to the start of the intervention to notify them that their calls could be recorded.

Calls from the recorded phone lines were initially screened based on length of call and excluded if less than 25 seconds. The remaining calls were reviewed three times by a member of the research team (RB), and were excluded if they did not concern an inpatient admission, involved admitting services other than IM, or the screener deemed the recording quality did not allow accurate evaluation (e.g., unintelligible or prematurely cut off). Eligible calls underwent stratified random sampling to achieve the pre-determined sample size. (See below.) We used sample stratification to ensure that the distribution of EP training level within the cohort of eligible calls was similar to the distribution in the final pre-/post-intervention samples. Recordings were de-identified and transcribed verbatim by a hospital-approved, independent third party.

#### Transcription Scoring

We created a 16-point scoring instrument reflecting the best practice recommendations used in creating SBAR-DR.^21^ Each element was scored as “communicated” or “not communicated,” based upon pre-defined requirements (Appendix). The scoring instrument was pilot tested on 15 sample cases, with revisions made based upon scorers’ feedback until consensus was reached.

Admission handoff transcripts were randomly assigned and independently scored by one of three dyads. Each of the dyads were comprised of one EP and one IM physician of similar training level to minimize the potential for undue influence. The dyads were blinded to physician- and patient-identifying information and whether the transcription was from the pre- or post-intervention group. Scoring disagreements were settled by consensus via in-person conference of dyad members.

The primary outcome was the composite admission handoff score (0–16 points), which was determined by summing the “communicated” elements of the transcribed verbal handoffs. Secondary outcomes included frequency of individual handoff elements; a global rating based on an anchored, five-point scale; and average length of handoff calls.

### Survey

We developed a post-intervention survey to assess EPs’ and IM physicians’ perceptions of the SBAR-DR strategy. Questions focused on patient safety and efficiency using a five-point, Likert-like scale. Before distribution, the survey was pilot tested for clarity and face-validity by two EPs and three IM physicians. A consent cover letter and link to an anonymous online survey was sent to eligible participants via their university email accounts. Participants who reported they had not participated in an ED admission handoff during the study period were excluded from the analysis.

### Analysis

Unpublished pilot data demonstrated that handoff scoring elements were communicated 30% of time. Anticipating a 25% absolute improvement,^6^ we determined that to achieve a 90% power with a significance level of 5% or less would require 87 pre-intervention and 87 post-intervention handoffs. Our estimation that up to 20% of calls might meet exclusion criteria during the scoring phase resulted in a final sample size of 110 pre- and 110 post-intervention admission handoffs.

We compared mean composite admission handoff quality scores and global rating scores using a t-test. Individual scoring elements were compared using chi-square tests. We calculated percent agreement and kappa statistics to determine inter-rater reliability for scoring elements. For the composite quality score and global rating scale, a general linear model was fit that included fixed-effect terms for time period (pre- or post-), EP training level, and the interaction of time period by training level. Type III tests were performed and, if significant, were followed by analysis of all possible pairwise comparisons of interest.

We calculated descriptive statistics for physician survey responses and handoff template use within the EHR. The method in which the written note was coded did not allow for analysis beyond descriptive terms and only produced data in the form of general use counts. Statistical calculations were completed using IBM SPSS v 22 and PC SAS version 9.4. We considered p-values of <0.05 statistically significant. This research project was approved for exempt status by the local institutional review board (#729-14-EX).

## RESULTS

**Figure 2:**
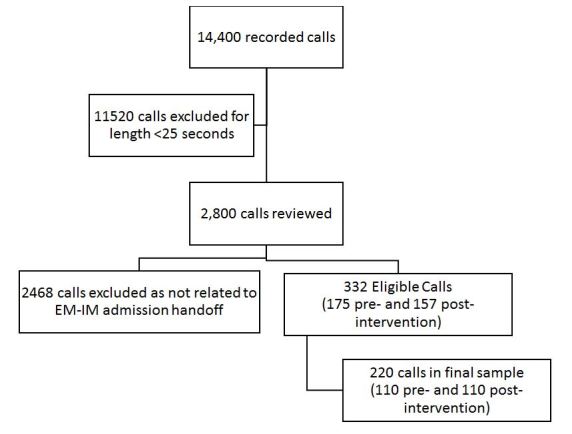
Study flow diagram for evaluation of admission-handoff recordings between emergency physicians and internal medicine physicians during the 120-day study period.

Approximately 14,400 calls took place on the recorded phone lines over the study period, with 20% lasting >25 seconds (Figure 2). After review, 332 calls (175 pre- and 157 post-intervention) met inclusion criteria, with 220 used in the final analysis (110 pre- and 110 post-intervention). Table 1 displays the handoff characteristics and admission-handoff quality scoring before and after introduction of SBAR-DR. For the primary outcome, there was a significant increase in composite quality score in the post-intervention recordings (mean 7.57 ± 2.42 vs. 8.45 ± 2.51, p=0.009). Individual content areas that showed improvement included opportunities to ask questions (70.6% vs. 82.7% p=0.034), agreement about disposition plan (41.8% vs. 62.7% p=0.002), and adherence to the SBAR-DR format (17.2% vs. 29.1% p= 0.038). There was a trend towards significance for stating severity of illness (7.3% vs 14.5% p=0.084), and use of closed-loop communication (27.3% vs. 38.2% p=0.085).

The inter-rater agreement for SBAR-DR scoring elements was moderate (0.41–0.60) to substantial (0.61–0.80) as measured by Cohen’s kappa.^26^ There was no significant difference in the global rating scale (2.95 +/− 0.85 vs. 3.09 +/− 0.85. p= 0.236). The mean handoff duration was longer in the post-intervention phase (2:15 minutes vs. 2:28 minutes, p=0.016). When analyzing scores based on EP training level, Postgraduate year 3 residents demonstrated significant improvement in composite quality scores (7.2, standard deviation [SD] 2.3 vs. 9.0, SD 2.3, p<0.01) and global rating score (2.8, SD 0.8 vs. 3.2, SD0.7, p=0.02). There were no statistically significant changes within other levels of training. The written handoff template was used for 51% of eligible admissions during the study period (329/642 admissions).

The post-intervention survey response rate was 50% for EPs (19/38) and 66% for IM physicians (20/30). Table 2 illustrates physicians’ perceptions of the SBAR-DR model. Overall, the majority of EPs and IM physicians felt that using SBAR-DR had a positive impact on patient safety and efficiency compared to prior handoff strategies.

## DISCUSSION

We found that the introduction of a standardized handoff process for patients being admitted from the ED to hospital setting resulted in improvements in verbal handoff quality. The driver of improvement was primarily due to improvements in opportunities for questions and reaching unambiguous agreement regarding patient disposition. Interactive questioning during handoffs is recommended by regulatory agencies^27^ and practice guidelines.^21^ Not only does this support clarifications and error-correction, but it also facilitates anticipatory guidance, reframing of the clinical picture, and creation of a shared mental model of patient care.^28^

Explicit agreement in disposition plan was also an important improvement. Uncertain assignment of responsibility is a known barrier to safe care transitions.^10^, ^13^ EPs and IM physicians are often uncertain when patient care is definitively transferred, especially for a patient boarding in the ED. This leaves the patient in limbo, during which time nursing and ancillary staff do not know where to direct concerns about changes in clinical trajectory or care needs. As part of our intervention, we explicitly tied the disposition decision and assignment of patient care, in which the admitting team assumed responsibility when an admission order was placed. This decision was then clearly delineated in the handoff template, where it was visible to all members of the patient care team.

As a result of using the SBAR-DR strategy, over half of surveyed physicians reported personally experiencing improved patient safety during the 60-day study period. Although the absolute improvement in quality scores was modest (approximately 12% improvement above baseline), the intervention resulted in the communication of approximately 100 pieces of additional information, any of which had the potential to improve the handoff process.

A recent survey of EM residency programs in the U.S. found poor adherence to standardized ED-to-inpatient handoff practices,^29^ and our study was no exception. In the post-intervention period, the SBAR-DR format was used for only 30% of verbal handoffs and the written template was used for 50%. The reason for this was likely multifactorial and related to both methodological and cultural barriers. Although the pilot study involved the institution’s largest admitting service, EPs performed admission handoffs with other admitting teams not included in the study. Having to shift between different handoff strategies may have limited EPs’ ability to acclimatize and integrate SBAR-DR into their daily practice. The adoption of the written handoff note also may have been hindered by the additional charting time required. Additionally, having fewer senior EM residents in the post-intervention cohort may have negatively impacted our post-intervention scores, as we found this group showed significant improvement in both handoff quality score and global rating scale. This supports prior research, which has found that residents’ ability to integrate handoff information may improve with experience.^30^

Additionally, handoff practices are an engrained part of a specialty’s culture. Although our study group included faculty and resident physician champions from IM and EM, we may not have fostered adequate buy-in from practicing providers to change practice routines. As institutions implement changes to inter-unit handoffs and care transitions, they need to address cultural complacency and build coalitions among affected members of the healthcare team.^31^ Possible solutions include inter-disciplinary communication training, which could give physicians an opportunity to practice standardized handoffs with one another, while also mitigating future conflicts via improved inter-personal engagement.^11^ Endorsement from senior physician leadership could also facilitate provider buy-in and adherence. Finally, the Joint Commission Center for Transforming Healthcare’s Targeted Solutions Tool® has shown promise in improving handoff communication by facilitating targeted needs assessment of local handoff practices, data collection, and quality improvement intervention.^32^

## LIMITATIONS

The study had several limitations. Implementation was conducted at a single institution, so results may not be generalizable to other settings. The pre/post study design cannot exclude the possibility that factors other than the intervention may have influenced the results. Since we scored written transcripts, we may have missed certain cues, such as voice inflection and tone, which can be important in verbal communication. Additionally, we used a novel scoring instrument, as we were unable to find a published, psychometrically-tested assessment instrument. Our scoring system was strict in its definitions of “communicated,” which may have biased results toward the null. Finally, the method in which the written note was coded in the EHR did not allow for analysis beyond descriptive counts.

## CONCLUSION

We found that introduction of a standardized admission-handoff process resulted in improved verbal handoff quality and that physicians felt it facilitated better patient safety and efficiency. Improvements may have been limited by inconsistent application of the SBAR-DR format. Future areas of study could include the institution-wide implementation of the SBAR-DR model to avoid the use of competing handoff strategies and efforts to better engage practicing physicians prior to implementation.

## Supplementary Information



## Figures and Tables

**Table 1:** Characteristics and content communication frequency before and after introduction of SBAR-DR^*^ admission handoff strategy.

Content	Before (n=110)	After (n=110)	p-value	Inter-rater Agreement (%)	Kappa Statistic
Characteristics					
EM level of training			0.061		
Faculty	11 (10%)	12 (11%)		NA	NA
PGY 3	56 (51%)	37 (34%)		NA	NA
PGY 2	15 (14%)	18 (16%)		NA	NA
PGY 1	28 (25%)	43 (39%)		NA	NA
Duration of handoff (min)	2:15	2:28	0.016		
SBAR-DR quality scoring					
Situation					
Reason for call, admission vs. consult	79 (71.8%)	82 (74.5%)	0.648	91.4	0.78
Working diagnosis	104 (94.5%)	105 (95.4%)	0.7571	93.6	0.43
Background					
Patient history	86 (78.2%)	82 (74.5%)	0.5256	88.2	0.68
Physical exam findings	39 (35.4%)	32 (29.1%)	0.3128	88.6	0.74
Test results	91 (82.7%)	95 (86.4%)	0.4556	92.3	0.68
Assessment					
Severity of illness	8 (7.3%)	16 (14.5%)	0.0836	91.4	0.55
Treatments performed in the ED	61 (55.4%)	71 (64.5%)	0.1688	91.4	0.82
Patient’s response to treatments in the ED	39 (35.4%)	43 (39.1%)	0.577	93.2	0.85
Degree of certainty in working diagnosis	84 (76.4%)	85 (77.3%)	0.8731	76.8	0.45
Risks and recommendations					
Pending tests or tasks	26 (23.6%)	35 (31.8%)	0.1753	90.9	0.75
Assignment of responsibility for pending tests or tasks	7 (6.4%)	12 (10.9%)	0.2301	92.7	0.52
Patient-specific risks that may impact care	39 (35.4%)	38 (34.5%)	0.8876	87.7	0.72
Discussion and disposition					
Opportunity for questions	77 (70.6%)	91 (82.7%)	0.0344	88.2	0.68
Disposition plan agreement	46 (41.8%)	69 (62.7%)	0.0019	88.2	0.76
Read-back					
Use of closed-loop communication	30 (27.3%)	42 (38.2%)	0.0847	86.8	0.68
SBAR-DR format followed	19 (17.3%)	32 (29.1%)	0.0378	87.3	0.63
Composite handoff quality score	7.57 (SD 2.42)	8.45 (SD 2.51)	0.0085	NA	NA
Global rating scale	2.955 + 0.850	3.091 + 0.852	0.236	68.2	0.61

*SBAR-DR*, Situation, Background, Assessment, Responsibilities & Risks, Discussion and Disposition, Read-back & Record; *PGY*, postgraduate year; *SD*, standard deviation, *ED*, emergency department; *EM*, emergency medicine.

**Table 2:** Survey results of emergency and internal medicine physicians’ perceptions of the SBAR-DR^*^ handoff strategy.

SBAR-DR strategy used for verbal handoff	Helpful	No effect don’t know	Harmful
How did SBAR-DR impact patient safety compared to prior handoff strategies?	61.6%	38.5%	0%
How did SBAR-DR impact efficiency of care compared to prior handoff strategies?	53.8%	35.9%	10.3%
What was the overall impact of SBAR-DR compared to prior handoff strategies?	61.5%	33.3%	5.2%
When the written handoff template was used during admission handoff, how did it impact patient safety compared to prior handoff strategies?	41%	56.4%	2.6%
	
Have you experienced a situation in which you feel patient safety was positively impacted because the SBAR-DR handoff strategy was used?	Yes 54.8%		No 45.2%

*SBAR-DR*, Situation, Background, Assessment, Responsibilities & Risks, Discussion and Disposition, Read-back & Record
