# Supplementary material for: Evaluation of a Novel Handoff Communication Strategy for Patients Admitted from the Emergency Department
Source: West J Emerg Med. 2018 Feb 8;19(2):372–9. doi: 10.5811/westjem.2017.9.35121 (PMC5851513; doi:10.5811/westjem.2017.9.35121)
Supplement: Supplementary file 1 [file wjem-19-372-s001.docx]

**Appendix: Quality Scoring Definitions for Admission Handoff Transcripts**

Instructions: For each transcript, score the content areas outlined below as "communicated" or "not communicated". To be counted as “communicated,” the content area must be specifically discussed according to the defined criteria.

| Situation: | Communicated (with or without prompting) = 1 | Not communicated = 0 |
| --- | --- | --- |
| 1. Admission vs. consult clearly stated | EM physician clearly states reason for call - admission vs consult. | Reason for call not clearly stated. E.g. "I got one for you" without explicit statement of purpose. |
| 1. Working diagnosis clearly stated | EM physician states their working diagnosis or reasoning for further inpatient evaluation. This includes lab abnormalities (hyperglycemia, elevated LFTs, etc.) or ruling-out life-threatening conditions (e.g. cardiac rule-out for chest pain), if it is explicitly stated as the reason for admission. | EM physician states patient chief complaint or presenting symptom (e.g. SOB), without further explanation of what they think is the underlying etiology or reason for further inpatient evaluation. |
| Background: |  |  |
| 1. Relevant patient history | States 3 or more of the following in relation to current patient problem: demographics, HPI, PMH/PSH, home medications, social history, family history. | Does not communicate required history. |
| 1. Discussion of physical exam findings | States both 1) one or more vital signs (acceptable to reference normal VS) AND 2) any other organ system (reference to normal exam findings counts). | Does not communicate required exam findings. |
| 1. Discussion of diagnostic test results | States results of any tests 2 performed in ED (lab, imaging, etc.). Acceptable to state that a result was normal/WNL. | Does not discuss at least 2 test results. |
| Assessment: |  |  |
| 1. Severity of illness | States severity of illness using following scale: can be seen 1) on the floor or stable 2) within the hour or "watcher" 3)ASAP or unstable. | Does not communicate severity of illness. |
| 1. Treatments performed in the ED | States any treatment performed in ED OR that no treatment given. E.g. IVF, pain medications, therapeutic procedures, etc. | Does not communicate treatments in the ED. |
| 1. Patient’s response to treatments in the ED | Clearly states how patient responded to treatments (either subjective or objective). | Does not communicate response to treatment. |
| 1. Discussion of degree of certainty in working diagnosis | Explicitly discusses any uncertainty in working diagnosis, other diagnoses considered in the differential, or clinical rationale to support or refute working diagnosis. | Does not discuss degree of certainty, other diagnoses considered, nor clinical rationale. |
| Risks/Recommendations: |  |  |
| 1. Pending tests/tasks | Lists pending tests/tasks OR that no pending tests/tasks are present. | Does not communicate if there are or are not pending tests/tasks. |

| Situation: | | Communicated (with or without prompting) = 1 | | Not communicated = 0 | | |
| --- | --- | --- | --- | --- | --- | --- |
| 1. Assignment of responsibility for pending tests/tasks | States who is responsible for following pending tests/tasks. | | | | Lists pending tests/tasks, but does not state who is responsible for follow-up. |  |
| 1. Patient-specific circumstances that may impact care | States any of the following patient-specific circumstance that may impact care: prolonged boarding, language barriers, psychiatric comorbidities, DNR/DNI code status, isolation requirements, significant social considerations OR that no such circumstances exist. | | | | Does not state if there are patient-specific circumstances. |  |
| Discussion/Disposition: |  | | | |  |  |
| 1. Questions | Any questions from admitting provider relevant to patient care OR opportunity for questions explicitly offered (e.g. Do you have any questions?). | | | | No questions asked and no opportunity offered. |  |
| 1. Disposition plan | Explicitly states disposition plan - bed request order, agreement to admit/consult, or plan to evaluate in ED prior to disposition decision being made. | | | | No explicit discussion of disposition plan. E.g. "We'll see him." or "We'll be down." would not count. |  |
| Responsibility of Care/Read-back: |  | | | |  |  |
| 1. Read-back | Uses of read-back to confirm disposition plan or assignment of patient care responsibilities. | | | | No use of read-back about elements listed. |  |
| 1. Did the handoff receiver and giver follow the SBAR-DR format | Discussion of at least one component in all 6 SBAR-DR areas and follows the general order outlined. | | | | Does not discuss elements in all 6 SBAR-DR areas OR does not follow any obvious formatting/order. |  |
|  | | | | | |  |
| Global rating scale (Scale 1-5) | | | | | |  |
| 1 = Poorly organized, rambling, or missing critical elements. Interaction was disrespectful or unproductive. Physicians did not develop shared plan of patient care. Patient safety may be at risk. | | | 3= Adequate, but contains some areas that are disorganized or non-critical elements are missing. Physicians develop implicit plan of care, but discussion is limited and closed-loop communication not utilized. | | 5= Well-organized, appropriately concise, and contains all the elements of a high-quality handoff. Communication is respectful and productive. Physicians create shared mental model of patient care, utilizing two-way discussion and closed-loop communication. |  |
